# Supplementary material for: Enhancing air quality predictions in Chile: Integrating ARIMA and Artificial Neural Network models for Quintero and Coyhaique cities
Source: PLoS One. 2025 Jan 10;20(1):e0314278. doi: 10.1371/journal.pone.0314278 (PMC11723610; doi:10.1371/journal.pone.0314278)
Supplement: Attachment — Submitted filename: Plos.pdf [file pone.0314278.s004.pdf]

Paper: Enhancing Air Quality Predictions in Chile: Integrating ARIMA and Artificial Neural Network Models for Quintero and Coyhaique Cities

Overall evaluation:

(1) Main contribution:
The authors focus on air quality predictions, employing a hybrid forecasting strategy (ARIMA, ANN). Their research is conducted in Chile (Quintero, Ventanas, Coyhaique I, and Coyhaique II). Model performance is evaluated based on metrics (AIC, Ljung-Box statistical tests, and diverse statistical indicators). The hybrid ARIMA-ANN models, enriched with external covariates like wind speed and direction, led to good predictive prowess ($R^2 > 0.90$). The dataset is significant, spanning five years (2016 – 2021). The source is the National Air Quality Information System from Chile (219 monitoring stations for meteorological variables and air quality).

(2) Defects of the paper:
   (a) The frequency of the data in your dataset for all analysed pollutants is not mentioned. For the sake of the reader, it should be added. Can you say something about the accuracy of the monitoring systems used by your National Air Quality Information System from Chile?
   (b) Some words are exaggerated ("demonstrated unparalleled predictive prowess"). I know papers that apply hybrid algorithms of ML found a better R^2. Indeed, they are not for Chile, but the idea is the same.
   (c) This study's limitations are not well emphasized. The same idea applies to the novelty elements.
   (d) A short description of the studied area and the climate from there is missing.
   (e) Conclusions are not clear enough at this moment. My suggestion is to use numbers for objectivity.

(3) Minor mistakes:
Figures 4, 5, and 6 do not have good resolution, but they might be connected to the PDF. Please check.

(4) Questions:
   (a) The author states: "Over the period from 2016 to 2021, the data available for PM2.5 and PM10 at the Coyhaique I monitoring station stood at 98.2% and 97.9%, respectively, while for Coyhaique II, these figures were 96.5% for PM2.5 and 96.0% for PM10. On the other hand, Quintero stations showed between 96.2% and 98.8 % of data availability." Do you suggest that there is missing data in the National Air Quality Information System from Chile? If the answer is positive, how many of them are in those time intervals chosen by you during Summer and Winter?
   (b) Why did you choose to analyse only PM2.5, PM10 and SO_2 as pollutants?
   (c) Why do you think that the proposed models perform differently in different locations?
   (d) How did you divide the dataset into training and validating datasets?

(5) Innovation elements: The discussions regarding the new findings can be improved. I suggest comparing their findings with other worldwide studies to emphasise the novelty elements. The authors provided an analysis of the potential of advanced technologies in refining air quality forecasts in Chile.

(6) Overall recommendation: With the identified issues addressed, the paper has the potential to be accepted after minor revisions. The authors are encouraged to consider the feedback provided and make the necessary adjustments to improve the clarity, significance, and objectivity of their paper.
