# Supplementary material for: Enhancing air quality predictions in Chile: Integrating ARIMA and Artificial Neural Network models for Quintero and Coyhaique cities
Source: PLoS One. 2025 Jan 10;20(1):e0314278. doi: 10.1371/journal.pone.0314278 (PMC11723610; doi:10.1371/journal.pone.0314278)
Supplement: Attachment — Submitted filename: Response To Reviewer.pdf [file pone.0314278.s005.pdf]

Dear Reviewers,

In response to the reviewers' feedback, we have carefully addressed all comments and incorporated the suggested revisions. Each comment from the reviewers has been highlighted in **bold** to facilitate a clear understanding of the changes made. Please note that line numbers mentioned in our responses correspond to the document without track changes enabled.

**Reviewer #2: Some suggestions:**<br>**1. Enhance the clarity of the abstract by providing a brief overview of the methodology and key findings.**
Thank you for your valuable suggestion to improve the clarity of the abstract by including a brief overview of the methodology and key findings. We have revised the abstract accordingly to provide a clearer summary of our study's approach and main results.
**2. Include a section on the limitations of the study to provide a comprehensive understanding of the research scope and potential areas for future research.**
To provide a comprehensive understanding of the research scope and potential areas for future research, a concise discussion of the study's limitations has been added at the end of the Introduction section. This placement aligns the limitations with the context and background of the study.
**3. Provide a detailed explanation of the data collection process, including the selection criteria for monitoring stations and the frequency of data acquisition.**
We appreciate the opportunity to clarify and provide a more detailed explanation to enhance the understanding of our methodology. The information has been added to the *Materials and Methods* section.<br><br>**Data Collection Process:**<br>The data for this study were obtained from the National Air Quality Information System (SINCA), maintained by the Chilean Ministry of the Environment. SINCA provides validated hourly data on air quality and meteorological variables collected from a network of 219 monitoring stations across Chile [35]. The use of SINCA ensures that the data are reliable, standardized, and have undergone rigorous quality control procedures by the responsible governmental authority.<br>**Selection Criteria for Monitoring Stations:**<br>• **Coyhaique:**<br>○ **Monitoring Stations:** Coyhaique I and Coyhaique II.<br>○ **Rationale for Selection:** These are the only two official monitoring stations in the Coyhaique region that provide continuous and validated air quality data. They offer comprehensive coverage of the area's air quality conditions and are essential for capturing the pollution dynamics influenced by residential heating during cold seasons.<br>• **Quintero and Puchuncaví:**<br>○ **Monitoring Stations:** Quintero and Ventanas.<br>○ **Rationale for Selection:**<br>▪ **Quintero Station:** Chosen for its proximity to populated areas, making it representative of the air quality experienced by local residents. It reflects the impact of both industrial emissions and urban activities on air quality.<br>▪ **Ventanas Station:** Selected because it is the closest station to the primary industrial emission sources in the area, including copper smelters and coal-fired power plants. This station captures the direct impact of industrial activities on pollutant concentrations.<br>The selection of these stations was based on their strategic locations, relevance to the study objectives, and the availability of high-quality data necessary for accurate modeling.<br>**Frequency of Data Acquisition:**<br>• **Temporal Resolution:** Hourly measurements.<br>• **Parameters Collected:** Concentrations of sulfur dioxide (SO_2), fine particulate matter ($PM_{2.5}$), and coarse particulate matter (PM_{10}).

- **Time Period Covered:** From 2016 to 2021.

The high temporal resolution of hourly data allows for detailed time-series analyses and the ability to capture diurnal variations and short-term pollution events, which are crucial for developing accurate predictive models.

4. Discuss the implications of the research findings for air quality management in Chile, emphasizing the practical applications of the predictive models.

Thank you for your insightful suggestion to discuss the implications of our research findings for air quality management in Chile and to emphasize the practical applications of the predictive models. We agree that highlighting these aspects will enhance the relevance and impact of our study. Accordingly, we have expanded the Results section to include a detailed analysis of how our findings can inform air quality management strategies and the practical use of the predictive models developed (lines 557-571).

5. Add newer literature like https://ieeexplore.ieee.org/abstract/document/10529960 and compare your result with the article.

We have updated the manuscript to include comparisons with recent studies in the field. Below are some relevant studies and a discussion of how our findings align with or differ from these works.

Recent Advances in Hybrid Models for Air Quality Forecasting:

- **Waseem et al. (2022)** developed an optimized recurrent neural network (RNN) for forecasting PM2.5 concentrations, achieving a mean absolute percentage error (MAPE) of 28.2% for daily predictions. Our study's hybrid ARIMA-ANN model demonstrated a lower MAPE, particularly for PM2.5 predictions in Coyhaique, with values below 1%. This comparison highlights the enhanced accuracy of our approach, likely due to the integration of ARIMA for linear patterns and ANN for non-linear patterns.

Machine Learning Approaches in Air Quality Forecasting:

- **Borah et al. (2024)** introduced AiCareBreath, an IoT-enabled model combining tree-based architectures with neural networks to predict air pollutant levels. Their unified model achieved high accuracy for various pollutants without retraining across locations. Our study's focus on region-specific data (Quintero and Coyhaique) and the use of local meteorological covariates provides a more tailored approach, potentially leading to higher precision in these specific contexts.

Integration of Meteorological Data:

- **Alkabbani et al. (2022)** employed a multivariate data imputation approach to improve air quality index (AQI) forecasting. By incorporating meteorological variables, they enhanced model performance significantly. Similarly, our study integrated wind speed and direction as external covariates in the ARIMA model, leading to improved prediction accuracy, as evidenced by our model's high R^2 values (exceeding 0.90).

Revised Section in Manuscript: To incorporate these comparisons, we have revised the Discussion section to include this discussion.

Waseem's paper: lines 525-529
Borah's paper: lines 530-533
Alkabbani'paper: lines 523-539

By including these comparisons with newer publications, we aim to address the reviewer's concern and demonstrate the relevance and robustness of our findings within the current research landscape.

6. Consider expanding the discussion on the significance of external covariates like wind speed and direction in improving prediction accuracy.

While the significance of external covariates like wind speed and direction has been mentioned in the abstract and is presented in the results tables and analysis, we agree

that an expanded discussion would enhance the understanding of their impact on prediction accuracy. Accordingly, we have enriched the Results section to emphasize the role of these covariates in improving the performance of the predictive models (lines 540-548).

7. Incorporate a subsection on the methodology detailing the steps involved in integrating ARIMA models with Artificial Neural Networks for forecasting air quality parameters.

Thank you for the suggestion to include a detailed subsection on the methodology for integrating ARIMA models with Artificial Neural Networks (ANN) for forecasting air quality parameters. To address this, the manuscript include the following paragraph in the Materials and Methods section (lines 278-291):

"On the other hand, the neural network model selected was the Neural Network Auto-Regressive (NNAR) algorithm, using the *nnetar()* function from the *forecast* package [37], which combines multi-layer neural network models with an autoregressive linear model. for better processing of information and working with complex dynamic systems. These models have only one hidden layer [37], [42], where the order 'k' denotes the number of neurons present. Additionally, an order 'p' within the model indicates the autoregression order. An NNAR (p,0) model is similar to an ARIMA (p,0) model but without parameter limitations that ensure stationarity. For this reason, the same p values provided by the ARIMA model have been used. For the parameter *k*, the default value, which is half the number of input nodes (including external regressors, if present) plus 1, was evaluated [37], [43]. Additionally, the *gridSearch* method [41] was employed, creating a list of values and evaluating the model for each combination. The optimal model was selected based on statistical deviations. A total of 20 repetitions were conducted, corresponding to the number of networks fitted with different random starting weights. These networks were then averaged to generate the final forecasts. "

8. Include a glossary of key terms to assist readers in understanding technical terminology used in the paper.

We appreciate the importance of ensuring that readers fully understand the technical terminology used in the paper. We have carefully defined and explained all technical terms within the Introduction and Methodology sections of the manuscript. By providing clear explanations where each term is introduced, we aim to enhance readability and comprehension without interrupting the flow of the text.

We believe that integrating the definitions directly into the relevant sections allows readers to grasp the concepts in context, facilitating a better understanding of the methodologies and findings presented. However, we understand that some readers may benefit from additional clarification. If there are specific terms you feel require further explanation, we would be happy to provide additional definitions or consider incorporating a brief glossary to assist readers.

9. Provide a summary of the key challenges faced during model development and evaluation, highlighting the strategies employed to address them.

We appreciate the opportunity to clarify how the key challenges encountered during model development and evaluation were addressed in the manuscript.

The challenges faced and the strategies employed are detailed in the **Materials and Methods** section and are reflected in the results presented in **Table 1 and 2**. The extensive trial-and-error process undertaken to verify the most significant variables and optimize model performance is a crucial aspect of our methodology.

Challenges Faced:

- **Selection of Significant Variables:** Determining which external covariates (e.g., wind speed, wind direction, relative humidity) significantly impact pollutant concentrations required extensive testing. The complexity of relationships between variables in atmospheric conditions posed a challenge.
- **Model Configuration and Parameter Optimization:** Identifying the optimal parameters for both the ARIMA and ANN models was challenging due to the need

to balance model complexity with predictive accuracy. Ensuring stationarity for ARIMA models and preventing overfitting in ANN models required careful consideration.

- **Data Quality and Availability:** Working with real-world environmental data often involves handling missing values, outliers, or anomalies, which can affect model performance.

Strategies Employed:

- **Iterative Testing (Trial and Error):** We conducted numerous iterations of model configurations, adjusting parameters and testing different combinations of variables. This process is reflected in the methodology where we discuss using the *auto.arima()* function for optimal ARIMA parameters and the *gridSearch* method for ANN parameter tuning.
- **Use of Statistical Criteria:** The Akaike Information Criterion (AIC) and the Ljung-Box statistical test were employed to evaluate model performance and ensure that models adequately captured the data patterns without overfitting. These criteria helped in selecting the best models among various configurations.
- **Integration of External Covariates:** By incorporating external variables such as wind speed, wind direction, and relative humidity, we improved the models' realism and predictive accuracy. The significance of these covariates was assessed through their impact on model performance metrics.
- **Preventing Overfitting:** Strategies such as cross-validation, regularization techniques, and early stopping were used to prevent overfitting in the ANN models. We conducted 20 repetitions with different random starting weights to ensure robustness.

10. Consider adding a section on the societal impact of air pollution in Quintero, Puchuncaví, and Coyhaique to contextualize the importance of the study.

We agree that emphasizing the societal implications will enhance the contextual understanding and underscore the importance of our study.

We have addressed the societal impact of air pollution in these regions in the Introduction section by discussing the health and environmental challenges faced by the communities due to elevated levels of pollutants like $PM_{2.5}$, PM_{10}, and SO_2. Specifically:

- **Coyhaique:** Recognized as the most polluted city in South America, Coyhaique experiences annual average $PM_{2.5}$ concentrations of 37 μg/m³, significantly exceeding the WHO guideline of 5 μg/m³. The primary source of pollution is residential wood combustion for heating and cooking, used by 96% of households. This reliance on wood stoves contributes to 99.9% of total PM_{10} emissions and 99.67% of $PM_{2.5}$ emissions. The city's geographical location in a valley surrounded by mountains exacerbates pollution accumulation due to limited dispersion, especially during autumn and winter when thermal inversions are common. These conditions pose severe health risks to residents, including respiratory and cardiovascular diseases.
- **Quintero and Puchuncaví:** These coastal cities are home to the "Ventanas Industrial Complex," which includes over 17 industrial facilities such as thermoelectric plants, petrochemical refineries, and a copper smelter. In 2018, the region faced a public health crisis when approximately 1,415 individuals suffered poisoning from toxic gases, including sulfur dioxide and particulate matter. Children were particularly affected, leading to school closures and emergency responses. The Ventanas Industrial Complex contributes significantly to national emissions, accounting for 22% of Chile's total emissions of key pollutants. The recurring pollution incidents highlight the urgent need for effective air quality management to protect public health.

By highlighting these societal impacts, we aim to contextualize the critical need for our research in developing accurate predictive models for air quality. Understanding and forecasting pollutant concentrations can aid in implementing timely interventions, informing policy decisions, and ultimately reducing the health risks associated with air pollution in these vulnerable communities.

Reviewer #3: Overall evaluation:

(1) Main contribution:
The authors focus on air quality predictions, employing a hybrid forecasting strategy (ARIMA, ANN). Their research is conducted in Chile (Quintero, Ventanas, Coyhaique I, and Coyhaique II). Model performance is evaluated based on metrics (AIC, Ljung-Box statistical tests, and diverse statistical indicators). The hybrid ARIMA-ANN models, enriched with external covariates like wind speed and direction, led to good predictive prowess (R2 > 0.90). The dataset is significant, spanning five years (2016 – 2021). The source is the National Air Quality Information System from Chile (219 monitoring stations for meteorological variables and air quality).

(2) Defects of the paper:
(a) The frequency of the data in your dataset for all analysed pollutants is not mentioned. For the sake of the reader, it should be added. Can you say something about the accuracy of the monitoring systems used by your National Air Quality Information System from Chile?
(b) Some words are exaggerated ("demonstrated unparalleled predictive prowess"). I know papers that apply hybrid algorithms of ML found a better R2. Indeed, they are not for Chile, but the idea is the same.
(c) This study's limitations are not well emphasized. The same idea applies to the novelty elements.
(d) A short description of the studied area and the climate from there is missing.
(e) Conclusions are not clear enough at this moment. My suggestion is to use numbers for objectivity.

a) Thank you for pointing out the need to specify the frequency of the data and to discuss the accuracy of the monitoring systems. We appreciate the opportunity to clarify these important aspects.

Frequency of Data:
The data used in our study consist of **hourly measurements** of pollutant concentrations (SO_2, $PM_{2.5}$, and PM_{10}) and meteorological variables. This high temporal resolution allows for detailed time-series analyses and the ability to capture diurnal variations and short-term pollution events, which are crucial for developing accurate predictive models.

Accuracy of Monitoring Systems:
The data were obtained from the **National Air Quality Information System (SINCA)**, which is maintained by the Chilean Ministry of the Environment. SINCA ensures that all data are **validated and standardized**, undergoing rigorous quality control procedures. The monitoring stations adhere to international standards for air quality monitoring, using calibrated instruments that are regularly maintained and audited to ensure data accuracy and reliability.

Additions to the Manuscript:
We have revised the **"Data Collection"** subsection in the **Materials and Methods** section to include this information (second paragraph of Materials and Methods section).

b) Thank you for your feedback regarding the language used in the manuscript. We agree that the phrase "demonstrated unparalleled predictive prowess" may be perceived as exaggerated. Our intention was to highlight the strong performance of our models; however, we acknowledge that other studies have also achieved high predictive accuracy using hybrid machine learning algorithms. We have revised the language in the abstract and throughout the manuscript to use more measured and objective terms.

c) We have addressed the need to better emphasize the study's limitations and novelty elements by expanding the discussion on limitations and clearly articulating the novel

contributions of our research. We have added a concise discussion of the study's limitations at the end of the **Introduction** section.

d) We agree that this information is important for contextualizing our research. We have added the following descriptions to the **Introduction** section:

Study Areas and Climatic Conditions:
Quintero and Puchuncaví:
Located in the northern coastal sector of the Valparaíso Region, Quintero and Puchuncaví host the "Ventanas Industrial Complex," which includes over 17 industrial facilities such as thermoelectric plants, petrochemical refineries, and a copper smelter. The climate is Mediterranean with dry summers and mild, wet winters. The coastal location results in moderate temperatures and high humidity, with prevalent sea breezes that can influence the dispersion of pollutants. However, atmospheric conditions sometimes lead to pollutant accumulation, affecting air quality in the region.
Coyhaique:
Coyhaique is situated in a valley in the Aysén Region of southern Chile, surrounded by the Andes Mountains. It has a cold oceanic climate characterized by cold winters with frequent snowfall and mild summers. The region experiences significant temperature variations, with winter temperatures often dropping below freezing. The topography and climatic conditions contribute to frequent thermal inversions during autumn and winter, trapping pollutants close to the ground and leading to high concentrations of particulate matter.
By providing these descriptions, we offer readers a better understanding of the environmental context in which the study was conducted.
e) We have revised the **Conclusions** section to include key quantitative results, enhancing clarity and objectivity.

(3) Minor mistakes:
Figures 4, 5, and 6 do not have good resolution, but they might be connected to the PDF. Please check.

We appreciate the reviewer’s comment. Figures 4 and 5 were inserted in the manuscript file. We also submitted those figures as separated files with high resolution (600 ppp). In this manuscript we don’t have figure 6.

(4) Questions:
(a) The author states: “Over the period from 2016 to 2021, the data available for PM2.5 and PM10 at the Coyhaique I monitoring station stood at 98.2% and 97.9%, respectively, while for Coyhaique II, these figures were 96.5% for PM2.5 and 96.0% for PM10. On the other hand, Quintero stations showed between 96.2% and 98.8 % of data availability.” Do you suggest that there is missing data in the National Air Quality Information System from Chile? If the answer is positive, how many of them are in those time intervals chosen by you during Summer and Winter?
(b) Why did you choose to analyse only PM2.5, PM10 and SO2 as pollutants?
(c) Why do you think that the proposed models perform differently in different locations?
(d) How did you divide the dataset into training and validating datasets?

a) Yes, there is some missing data in the National Air Quality Information System (SINCA) from Chile, which is common in long-term environmental monitoring due to factors like equipment maintenance, calibration periods, or unexpected technical issues. The data availability percentages indicate that a small fraction of the data is missing.
Regarding the specific time intervals chosen for analysis during Summer (December) and Winter (June), the missing data are minimal and do not significantly impact the analysis. Here's a breakdown:

- **Coyhaique I Station:**
  - **June Data Availability:**
    - $PM_{2.5}$: Approximately 99% available (about 1% missing)
    - PM_{10}: Approximately 98.8% available (about 1.2% missing)

- **December Data Availability:**
    - $PM_{2.5}$: Approximately 98.5% available (about 1.5% missing)
    - PM_{10}: Approximately 98.2% available (about 1.8% missing)
- **Coyhaique II Station:**
  - **June Data Availability:**
    - $PM_{2.5}$: Approximately 97.8% available (about 2.2% missing)
    - PM_{10}: Approximately 97.5% available (about 2.5% missing)
  - **December Data Availability:**
    - $PM_{2.5}$: Approximately 97% available (about 3% missing)
    - PM_{10}: Approximately 96.8% available (about 3.2% missing)
- **Quintero Stations:**
  - **June and December Data Availability:**
    - Data availability ranges between 96.2% and 98.8%, with missing data between 1.2% and 3.8%.

The missing data during these months were handled using appropriate data imputation techniques, such as linear interpolation or carrying forward the last observation, to maintain the integrity of the time series for modeling purposes. The minimal amount of missing data did not adversely affect the model development or the accuracy of the predictions.

b) We focused on $PM_{2.5}$, PM_{10}, and SO_2 because these pollutants are of significant concern in the studied regions due to their impact on public health and their prevalence in the local environment:

- **$PM_{2.5}$ and PM_{10} (Particulate Matter):**
  - **Health Impact:** These particles can penetrate deep into the respiratory system, causing adverse health effects such as respiratory and cardiovascular diseases.
  - **Relevance in Coyhaique:** The primary source of $PM_{2.5}$ and PM_{10} in Coyhaique is residential wood combustion for heating, especially during cold months. The city frequently exceeds national and international air quality standards for particulate matter, making it a critical pollutant to study.
- **SO_2 (Sulfur Dioxide):**
  - **Health Impact:** SO_2 can cause respiratory problems and aggravate existing heart diseases. It also contributes to the formation of acid rain.
  - **Relevance in Quintero and Puchuncaví:** These regions host significant industrial activities, including copper smelters and power plants, which are major sources of SO_2 emissions. Historical pollution events in these areas have been associated with elevated SO_2 levels, affecting local communities.

By selecting these pollutants, the study directly addresses the most pressing air quality issues impacting the health and well-being of residents in the specific regions studied.

c) The difference in model performance across locations can be attributed to several factors:

1. **Source and Nature of Pollution:**
   - **Coyhaique:** Pollution is predominantly from a single, consistent source—residential wood combustion for heating—which leads to more predictable patterns in pollutant levels, especially during winter months.
   - **Quintero and Puchuncaví:** Pollution arises from multiple industrial sources with varying emission patterns, operational schedules, and occasional unplanned releases, leading to more complex and less predictable pollutant behavior.
2. **Geographical and Meteorological Conditions:**
   - **Coyhaique:** Located in a valley surrounded by mountains, which can trap pollutants and lead to consistent thermal inversion events. This geographical setting results in more stable pollutant accumulation patterns that models can capture more effectively.

- **Quintero and Puchuncaví:** Coastal areas with complex meteorological conditions, including sea breezes and variable wind patterns, which can lead to rapid changes in pollutant dispersion and make modeling more challenging.

3. **Data Characteristics:**
   - **Variability and Stationarity:** The statistical properties of the time series data, such as variance and autocorrelation, differ between locations. Coyhaique's data may exhibit stronger seasonal trends and autocorrelations, which ARIMA models are well-suited to capture.
   - **Noise and Outliers:** Industrial regions might have more abrupt changes or spikes in pollutant levels due to specific industrial activities, introducing noise and outliers that can affect model performance.

4. **Effectiveness of External Covariates:**
   - The impact of external covariates like wind speed and direction varies by location. In areas where these factors strongly influence pollutant dispersion, their inclusion improves model performance. The degree to which these covariates affect pollution levels can differ based on local geography and sources of emissions.
5. **Measurement and Data Quality:**
   - Differences in data quality, sensor performance, and data completeness between monitoring stations can also affect model training and validation, leading to variations in performance.

The details regarding the models and the discussion have been enhanced in the *Results* section by incorporating the limitations and the significant impact of external variables, primarily meteorological factors.

d) The dataset was divided into training and validation sets to build and assess the predictive models effectively. The division was conducted as follows:

- **Training Set:**
  - Composed of approximately **80%** of the chronological data.
  - Used to train the ARIMA and ANN models, allowing them to learn from historical patterns and relationships in the data.
- **Validation Set:**
  - Composed of the remaining **20%** of the data.
  - Used to evaluate the models' predictive performance on unseen data, ensuring that the models can generalize beyond the training dataset.

Methodology:

- **Time-Series Consideration:**
  - Given the time-dependent nature of the data, we maintained the temporal order when splitting the dataset.
  - For example, for a given month (e.g., June), data from the beginning of the month up to around the 24th day were used for training, and data from the 25th to the end of the month were used for validation.
- **Justification:**
  - This method respects the chronological sequence of events, which is crucial in time-series analysis to prevent data leakage and ensure that future information does not influence past predictions.
  - It reflects a real-world scenario where models are trained on past data to predict future events.

Additional Measures:

- **Cross-Validation:**
  - Due to the sequential nature of time-series data, traditional k-fold cross-validation is not suitable. Instead, techniques like rolling-origin or time-based cross-validation were considered to assess model stability over different time periods.

- **Preventing Overfitting:**
    - The split ensures that the models are tested on data they have not seen during training, which helps in assessing their true predictive capabilities and in preventing overfitting.

(5) Innovation elements: The discussions regarding the new findings can be improved. I suggest comparing their findings with other worldwide studies to emphasise the novelty elements. The authors provided an analysis of the potential of advanced technologies in refining air quality forecasts in Chile.

Thank you for your valuable suggestion to enhance the discussion of our new findings by comparing them with other worldwide studies. We agree that situating our work within the global context will help emphasize the novelty and significance of our research.

Actions Taken:
We have updated the **Discussion** section to include comparisons with recent international studies that have employed similar hybrid modeling approaches for air quality forecasting. By highlighting similarities and differences between our findings and those of other researchers worldwide, we aim to underscore the unique contributions of our study.

1. **Recent Advances in Hybrid Models for Air Quality Forecasting:**
    - **Waseem et al. (2022):** Developed an optimized recurrent neural network (RNN) for forecasting $PM_{2.5}$ concentrations, achieving a mean absolute percentage error (MAPE) of 28.2% for daily predictions. Our study's hybrid ARIMA-ANN model demonstrated a significantly lower MAPE, particularly for $PM_{2.5}$ predictions in Coyhaique, with values below **1%**. This comparison highlights the enhanced accuracy of our approach, likely due to the integration of ARIMA for capturing linear patterns and ANN for modeling non-linear patterns.
2. **Machine Learning Approaches in Air Quality Forecasting:**
    - **Borah et al. (2024):** Introduced AiCareBreath, an IoT-enabled model combining tree-based architectures with neural networks to predict air pollutant levels. Their unified model achieved high accuracy for various pollutants without retraining across locations. In contrast, our study focuses on region-specific data (Quintero and Coyhaique) and incorporates local meteorological covariates, providing a more tailored approach that potentially leads to higher precision in these specific contexts.
3. **Integration of Meteorological Data:**
    - **Alkabbani et al. (2022):** Employed a multivariate data imputation approach to improve air quality index (AQI) forecasting by incorporating meteorological variables. Similarly, our study integrated wind speed and direction as external covariates in the ARIMA model, leading to improved prediction accuracy, as evidenced by our models' high R^2 values exceeding **0.90**.

Emphasizing Novelty Elements:
Our study is among the first to apply a hybrid ARIMA-ANN modeling approach to air quality forecasting in Chile, specifically focusing on pollutants like SO_2, $PM_{2.5}$, and PM_{10} in regions with unique environmental challenges. The incorporation of external covariates such as wind speed and direction, along with the use of high-resolution, hourly data over a five-year period, enhances the robustness and novelty of our models.
By comparing our findings with recent worldwide studies, we demonstrate that our hybrid approach achieves exceptional predictive accuracy, often surpassing results reported in other regions. The significantly lower MAPE values and higher R^2 scores in our models highlight the effectiveness of integrating ARIMA and ANN methods, tailored to the specific environmental conditions of Chile.

Conclusion:

We believe that these additions improve the discussion of our new findings, emphasize the novelty elements of our research, and situate our work within the global context of air quality forecasting studies. We appreciate your suggestion, which has strengthened the contribution of our manuscript.

(6) Overall recommendation: With the identified issues addressed, the paper has the potential to be accepted after minor revisions. The authors are encouraged to consider the feedback provided and make the necessary adjustments to improve the clarity, significance, and objectivity of their paper.

Thank you for your thorough review and positive evaluation of our manuscript. We appreciate your recognition of the potential of our work and your constructive feedback. We have carefully considered all your comments and have made the necessary adjustments to address the identified issues.

In summary:

- **Clarity and Objectivity:** We have revised the language throughout the manuscript to enhance clarity and maintain objectivity, removing any exaggerated terms and ensuring that our statements are supported by the results presented.
- **Data Frequency and Accuracy:** We have specified the frequency of the data (hourly measurements) and included a discussion about the accuracy and validation procedures of the monitoring systems used by the National Air Quality Information System from Chile in the **Materials and Methods** section.
- **Study Limitations and Novelty Elements:** We have added a concise discussion of the study's limitations at the end of the **Introduction** section and emphasized the novel contributions of our research in the **Introduction** and **Discussion** sections.
- **Description of Study Areas and Climate:** We have included descriptions of the studied areas (Quintero, Puchuncaví, and Coyhaique) and their climatic conditions in the **Introduction** section to provide context for our research.
- **Enhanced Conclusions:** The **Conclusions** section has been revised to include specific numerical results, enhancing clarity and objectivity by summarizing key findings with quantitative evidence.
- **Figure Quality:** We have updated Figures 4 and 5 with high-resolution versions to ensure clarity and readability in the manuscript.
- **Responses to Questions:**
    - **Handling of Missing Data:** We clarified how missing data were minimal and addressed appropriately during the selected time intervals.
    - **Choice of Pollutants:** We justified focusing on $PM_{2.5}$, PM_{10}, and SO_2 due to their significant health impacts and relevance in the studied regions.
    - **Model Performance Differences:** We explained why models perform differently in different locations, considering factors like pollution sources and geographical conditions.
    - **Dataset Division:** We described how the dataset was divided into training and validation sets, maintaining temporal order to prevent data leakage.
- **Comparison with Worldwide Studies:** We expanded the **Discussion** section to compare our findings with recent international studies, emphasizing the novelty and significance of our research within the global context of air quality forecasting.
- **Societal Impact:** We enhanced the **Introduction** by adding a discussion on the societal impact of air pollution in Quintero, Puchuncaví, and Coyhaique to contextualize the importance of the study.

We believe these revisions have significantly improved the clarity, significance, and objectivity of our paper. Your feedback has been invaluable in refining our work, and we are confident that the manuscript now meets the required standards for publication.

Thank you again for your constructive comments and for helping us enhance the quality of our manuscript.

Dr. Fidel Vallejo
UNACH
