# Supplementary material for: Enhancing air quality predictions in Chile: Integrating ARIMA and Artificial Neural Network models for Quintero and Coyhaique cities
Source: PLoS One. 2025 Jan 10;20(1):e0314278. doi: 10.1371/journal.pone.0314278 (PMC11723610; doi:10.1371/journal.pone.0314278)
Supplement: Attachment — Submitted filename: Rebutal letter.docx [file pone.0314278.s001.docx]

Dear Academic Editor and Reviewers,

This document details the changes made to address the observations and suggestions provided for the manuscript. We deeply appreciate the time you have dedicated to reviewing the document.

Best regards,

Dr. Fidel Vallejo

**Journal requeriments**

**1. Please ensure that your manuscript meets PLOS ONE's style requirements, including those for file naming. The PLOS ONE style templates can be found at**
[**https://journals.plos.org/plosone/s/file?id=wjVg/PLOSOne_formatting_sample_main_body.pdf**](https://journals.plos.org/plosone/s/file?id=wjVg/PLOSOne_formatting_sample_main_body.pdf) **and**
[**https://journals.plos.org/plosone/s/file?id=ba62/PLOSOne_formatting_sample_title_authors_affiliations.pdf**](https://journals.plos.org/plosone/s/file?id=ba62/PLOSOne_formatting_sample_title_authors_affiliations.pdf)

We have thoroughly reviewed the document and are pleased to confirm that it adheres to PLOS ONE's style requirements.

**2. Please note that PLOS ONE has specific guidelines on code sharing for submissions in which author-generated code underpins the findings in the manuscript. In these cases, all author-generated code must be made available without restrictions upon publication of the work. Please review our guidelines at** [**https://journals.plos.org/plosone/s/materials-and-software-sharing#loc-sharing-code**](https://journals.plos.org/plosone/s/materials-and-software-sharing#loc-sharing-code) **and ensure that your code is shared in a way that follows best practice and facilitates reproducibility and reuse.**

We appreciate the reminder regarding PLOS ONE's guidelines on code sharing. In our manuscript, the code was generated using R programming language, and we have taken the necessary steps to ensure its accessibility and reproducibility. Specifically, we have uploaded all author-generated code to a publicly accessible repository to comply with the journal's requirements and facilitate transparency and reuse of our methods (DOI: 10.34740/kaggle/ds/4949501). You can find the code repository linked in the manuscript for complete availability and ease of access. We are committed to following best practices in code sharing to support reproducibility and transparency in scientific research.

**3. Please expand the acronym “DICYT” (as indicated in your financial disclosure) so that it states the name of your funders in full.
This information should be included in your cover letter; we will change the online submission form on your behalf.**

The information was included in the cover letter.

**4. Thank you for stating the following in your Competing Interests section:
"No"**

**Please complete your Competing Interests on the online submission form to state any Competing Interests. If you have no competing interests, please state "The authors have declared that no competing interests exist.", as detailed online in our guide for authors at** [**http://journals.plos.org/plosone/s/submit-now**](http://journals.plos.org/plosone/s/submit-now) **This information should be included in your cover letter; we will change the online submission form on your behalf.**

The authors have declared that no competing interests exist

**5. We note that your Data Availability Statement is currently as follows: [All relevant data are within the manuscript and its Supporting Information files.]**

**Please confirm at this time whether or not your submission contains all raw data required to replicate the results of your study. Authors must share the “minimal data set” for their submission. PLOS defines the minimal data set to consist of the data required to replicate all study findings reported in the article, as well as related metadata and methods (**[**https://journals.plos.org/plosone/s/data-availability#loc-minimal-data-set-definition**](https://journals.plos.org/plosone/s/data-availability#loc-minimal-data-set-definition)**).**

**For example, authors should submit the following data:**

**- The values behind the means, standard deviations and other measures reported;
- The values used to build graphs;
- The points extracted from images for analysis.**

**Authors do not need to submit their entire data set if only a portion of the data was used in the reported study.**

**If your submission does not contain these data, please either upload them as Supporting Information files or deposit them to a stable, public repository and provide us with the relevant URLs, DOIs, or accession numbers. For a list of recommended repositories, please see** [**https://journals.plos.org/plosone/s/recommended-repositories**](https://journals.plos.org/plosone/s/recommended-repositories)**.**

**If there are ethical or legal restrictions on sharing a de-identified data set, please explain them in detail (e.g., data contain potentially sensitive information, data are owned by a third-party organization, etc.) and who has imposed them (e.g., an ethics committee). Please also provide contact information for a data access committee, ethics committee, or other institutional body to which data requests may be sent. If data are owned by a third party, please indicate how others may request data access.**

Thank you for bringing this to our attention. We confirm that the raw data necessary to replicate the results of our study have been made available. Specifically, the data used to generate the graphs in our manuscript have been uploaded to a publicly accessible repository on Kaggle (10.34740/kaggle/ds/4949501). Additionally, the data from the monitoring stations utilized in our study are publicly available on the website https://sinca.mma.gob.cl/. We have ensured that all relevant data, including the values behind the means, standard deviations, and other measures reported, as well as the values used to build graphs, have been included in the repository. Therefore, researchers interested in replicating our findings can access the complete dataset and metadata necessary for reproducibility. If you require further information or have any additional questions regarding data access, please do not hesitate to contact us. We are committed to transparency and reproducibility in our research practices and are happy to assist with any inquiries regarding data availability.

The following text have been added in the caption: Base map and data from OpenStreetMap and OpenStreetMap Foundation.

**6. We note that [Figure 3] in your submission contain [map/satellite] images which may be copyrighted. All PLOS content is published under the Creative Commons Attribution License (CC BY 4.0), which means that the manuscript, images, and Supporting Information files will be freely available online, and any third party is permitted to access, download, copy, distribute, and use these materials in any way, even commercially, with proper attribution. For these reasons, we cannot publish previously copyrighted maps or satellite images created using proprietary data, such as Google software (Google Maps, Street View, and Earth). For more information, see our copyright guidelines:** [**http://journals.plos.org/plosone/s/licenses-and-copyright**](http://journals.plos.org/plosone/s/licenses-and-copyright)**.

We require you to either (1) present written permission from the copyright holder to publish these figures specifically under the CC BY 4.0 license, or (2) remove the figures from your submission:

a. You may seek permission from the original copyright holder of Figure 3 to publish the content specifically under the CC BY 4.0 license.

We recommend that you contact the original copyright holder with the Content Permission Form (**[**http://journals.plos.org/plosone/s/file?id=7c09/content-permission-form.pdf**](http://journals.plos.org/plosone/s/file?id=7c09/content-permission-form.pdf)**) and the following text:
“I request permission for the open-access journal PLOS ONE to publish XXX under the Creative Commons Attribution License (CCAL) CC BY 4.0 (**[**http://creativecommons.org/licenses/by/4.0/**](http://creativecommons.org/licenses/by/4.0/)**). Please be aware that this license allows unrestricted use and distribution, even commercially, by third parties. Please reply and provide explicit written permission to publish XXX under a CC BY license and complete the attached form.”

Please upload the completed Content Permission Form or other proof of granted permissions as an "Other" file with your submission.

In the figure caption of the copyrighted figure, please include the following text: “Reprinted from [ref] under a CC BY license, with permission from [name of publisher], original copyright [original copyright year].”

b. If you are unable to obtain permission from the original copyright holder to publish these figures under the CC BY 4.0 license or if the copyright holder’s requirements are incompatible with the CC BY 4.0 license, please either i) remove the figure or ii) supply a replacement figure that complies with the CC BY 4.0 license. Please check copyright information on all replacement figures and update the figure caption with source information. If applicable, please specify in the figure caption text when a figure is similar but not identical to the original image and is therefore for illustrative purposes only.
The following resources for replacing copyrighted map figures may be helpful:

USGS National Map Viewer (public domain):** [**http://viewer.nationalmap.gov/viewer/**](http://viewer.nationalmap.gov/viewer/)
**The Gateway to Astronaut Photography of Earth (public domain):** [**http://eol.jsc.nasa.gov/sseop/clickmap/**](http://eol.jsc.nasa.gov/sseop/clickmap/)
**Maps at the CIA (public domain):** [**https://www.cia.gov/library/publications/the-world-factbook/index.html**](https://www.cia.gov/library/publications/the-world-factbook/index.html) **and** [**https://www.cia.gov/library/publications/cia-maps-publications/index.html**](https://www.cia.gov/library/publications/cia-maps-publications/index.html)
**NASA Earth Observatory (public domain):** [**http://earthobservatory.nasa.gov/**](http://earthobservatory.nasa.gov/)
**Landsat:** [**http://landsat.visibleearth.nasa.gov/**](http://landsat.visibleearth.nasa.gov/)
**USGS EROS (Earth Resources Observatory and Science (EROS) Center) (public domain):** [**http://eros.usgs.gov/**](http://eros.usgs.gov/)**#
Natural Earth (public domain):** [**http://www.naturalearthdata.com/**](http://www.naturalearthdata.com/)

We have replaced Figure 3 with a map image sourced from OpenStreet Maps, which is licensed under the CC BY 4.0 license. As such, the new figure complies with the licensing requirements of PLOS ONE, allowing for unrestricted use and distribution, even commercially, by third parties. Regarding the polygons related to the boundary lines of countries, states (regions in the case of Chile), and provinces, they come from the Natural Earth Data source, coinciding with one of the recommended sources previously mentioned.

**Reviewer #1: I have reviewed the manuscript "Enhancing Air Quality Predictions in Chile: Integrating ARIMA and Artificial Neural Network Models for Quintero and Coyhaique Cities" Before accept the manuscript kindly reply the below quires.
a) Why only two cities are chosen as sample sites ?**

In Chile, the main pollution problems are associated with the presence of industrial parks in the so-called "sacrifice zones," such as Mejillones and Quintero-Puchuncaví, which have significant SO2 emissions, even causing episodes of mass poisoning in the population. On the other hand, there are issues related to the presence of particulate matter due to the combustion of wood, pellets, and other biomass for heating and cooking, mainly in the southern part of the country. In this regard, Coyhaique represents these problems prominently, having been classified as the most polluted city in the Americas. Therefore, the objective of this study was to evaluate air quality prediction models in these two geographical areas, which reflect the main challenges of air pollution in Chile. This justification is detailed in the Introduction section of the manuscript, where the serious problem of Chile's industrial and residential areas is described, along with the country's complex geography influenced by the presence of the Andes and coastal mountain ranges.

**b) Kindly elaborate the methodology section of both ARIMA and ANN.**

The methodology section detailing both the ARIMA and ANN approaches has been comprehensively incorporated into the manuscript, outlining the criteria utilized in the study. In the Methodology section, detailed explanations of the procedures followed for both ARIMA and ANN modeling have been provided, including parameter selection, model development, and evaluation techniques. The ARIMA methodology encompasses the identification of parameters using auto.arima(), graphical analysis of time series data, and the application of statistical tests such as the Akaike Information Criterion (AIC) and Ljung-Box test. Additionally, for ANN modeling, the Neural Network Auto-Regressive (NNAR) algorithm has been selected, incorporating a combination of multilayer neural networks with an autoregressive linear model to process information effectively. The process of parameter selection for ANN models involves methods such as grid search, where a range of values is evaluated to determine the optimal combination based on statistical deviations. Furthermore, the methodology section emphasizes the integration of external covariates, such as meteorological data and pollutant concentrations, to enhance the predictive capabilities of both ARIMA and ANN models. Overall, the methodology section provides a detailed overview of the approaches utilized, ensuring transparency and reproducibility in the study's methods.

**c) There are many models to predict the future phenomena like ANFIS, Tree models, optimization, AR, ARMA, MA, ML, AI, coupled etc models, why you select only these two models?**

The selection of ARIMA and ANN models was driven by their proven effectiveness in time series forecasting, including air quality prediction, as well as their compatibility with our research objectives. By integrating these two methodologies, we aimed to leverage their complementary strengths in capturing both linear trends and nonlinear relationships present in air quality data. Previous research has demonstrated the competitive performance of ARIMA and ANN models in air quality forecasting, and their compatibility with available data and resources further supported their selection for our study. While we acknowledge the existence of alternative modeling approaches, such as ANFIS, Tree models, and optimization techniques, our decision was guided by the established effectiveness and suitability of ARIMA and ANN models for our research objectives. We remain open to exploring alternative techniques in future research endeavors as warranted by the research context and objectives.

**d) Conclusion section must be in point wise outcome of the current research.**

The conclusion section has been extensively revised to reflect the outcomes of the current research, incorporating insights from the newly added references and addressing the inherent limitations of mathematical models. The revised conclusion presents a comprehensive overview of the study findings, highlighting key insights gleaned from the analysis of air quality dynamics in Chilean cities, particularly Quintero and Coyhaique. Firstly, the conclusion underscores the efficacy of the hybrid ARIMA-ANN modeling approach in improving air quality predictions, as evidenced by the rigorous evaluation of model performance metrics such as R2, RMSE, and MAPE. The integration of external covariates, including meteorological data and pollutant concentrations, has further enhanced the predictive accuracy of the models, enabling more reliable forecasts of sulfur dioxide (SO2), and coarse particulate matter (PM10). Moreover, the conclusion acknowledges the complex interplay of geographical and climatic factors on air quality dynamics, particularly in regions characterized by industrial activities and biomass combustion for heating. The study findings emphasize the significance of region-specific strategies for effective air quality management, highlighting the need for tailored interventions to mitigate pollution sources and improve environmental health outcomes. Furthermore, the conclusion reflects on the broader implications of the research findings for environmental policy and public health initiatives. By providing valuable insights into the factors influencing air quality dynamics, the study contributes to the development of evidence-based strategies for mitigating air pollution and safeguarding public health. Additionally, the conclusion underscores the importance of continued research efforts to refine modeling approaches and enhance the accuracy of air quality predictions, thereby facilitating informed decision-making and proactive interventions to address environmental challenges.

**e) Some more recent references should be added.**

Additional recent references have been incorporated into both the introduction and results sections of the manuscript. These new sources enrich the discussion by providing updated insights and perspectives on air quality dynamics, enhancing the relevance and comprehensiveness of the study.

**Reviewer #2: The contribution of "Integrated ARIMA and ANN models for accurate air quality predictions" lies in its approach of combining two well-established methods, AutoRegressive Integrated Moving Average (ARIMA) and Artificial Neural Networks (ANN), to improve air quality predictions.
1. Evaluation Metrics: It would be beneficial to employ a variety of evaluation metrics to assess the performance of the integrated ARIMA and ANN models comprehensively. Metrics such as mean absolute error (MAE), root mean square error (RMSE), and correlation coefficients can provide insights into different aspects of model performance.**

We sincerely appreciate your attention and valuable feedback on our article "Integrated ARIMA and ANN models for accurate air quality predictions." We especially value your suggestion to employ a variety of evaluation metrics for a more comprehensive understanding of the performance of the integrated ARIMA and ANN models.

We are pleased to inform you that, as you mentioned, we have already included a detailed description of the evaluation metrics used, such as mean absolute error (MAE), root mean square error (RMSE), and the coefficient of correlation (R2), at the end of our methodology section. Additionally, we have presented the results of our statistical analysis in Table 2, specifically highlighting these three evaluators. We firmly believe that this selection of metrics provides a thorough and meaningful assessment of our models' performance.

**2. Parameter Optimization: Fine-tuning the ARIMA and ANN models' parameters could improve prediction accuracy. Optimization techniques such as grid search or evolutionary algorithms can be employed to find the optimal set of parameters for the integrated model.**

The methodology has been expanded to provide a more detailed explanation of the criteria followed for selecting the appropriate parameters for both the ARIMA and ANN models. We have incorporated an additional section where we describe the approaches used to tune the model parameters, highlighting optimization techniques such as grid search and evolutionary algorithms. This expansion of the methodology provides greater transparency and clarity on the parameter selection process, enabling us to enhance the accuracy of our air quality predictions.

**3. Feature Engineering: Exploring additional input features or incorporating domain knowledge into the modeling process may enhance the predictive capabilities of the integrated models. For example, meteorological data or data from other environmental sensors could be included to capture more comprehensive influences on air quality.**

In our study, meteorological variables are already included as input features in the models. These variables were directly obtained from observations at the monitoring stations analyzed in the study. Therefore, we have already considered the influence of meteorological factors on air quality predictions as part of our modeling process.

**4. The novelty of the work depends on the specific context and the extent to which similar approaches have been explored in the literature. If integrating ARIMA and ANN models for air quality prediction is relatively uncommon or if the study introduces novel methodologies or insights, then the work can be considered novel. However, the novelty may be limited if similar integrated approaches have been extensively explored in the literature. A thorough literature review and comparison with existing studies can help determine the level of novelty in the work.**

The validity of our approach has been demonstrated through rigorous statistical analysis, and its novelty has been validated through a thorough review of existing literature, particularly in countries with high pollution levels such as India and China. Our study reveals that integrating ARIMA and ANN models for air quality prediction is not only feasible but also effective in addressing prediction challenges posed by complex meteorological and topographical conditions. Furthermore, our research emphasizes the novelty of our study in the Chilean context. It represents the first attempt to compare two types of pollution prevalent in Chile: industrial pollution, dominated by SO2 emissions from copper concentrate processing, and PM10 pollution resulting from biomass and wood pellet burning. This unique focus on comparing different sources of pollution in Chilean cities adds significant value to the existing literature and provides insights into the specific challenges faced by the country in managing air quality.

**5. Potential Improvement: Spatially Explicit Modeling
The authors could consider employing spatially explicit modeling approaches to account for the heterogeneous distribution of pollutants influenced by Chile's unique topography. These methods allow for incorporating geographical features and terrain characteristics into air quality models, providing a more realistic representation of pollutant dispersion patterns. Additionally, integrating data from satellite imagery or remote sensing technologies can further enhance the spatial resolution of the modeling framework.**

In our study, we have carefully analyzed over five years of data to establish representative pollution patterns, selecting two distinct zones for model validation. One zone is an industrial area, characterized by significant industrial activity and predominant SO2 emissions, located in central Chile along the coastal region. The other zone is a commercial-residential area situated in southern Chile, characterized by the burning of pellets and biomass during winter, leading to elevated PM10 levels that exceed regulatory standards. This area has been designated as saturated with PM10 pollution. While spatially explicit modeling approaches may offer insights into pollutant dispersion patterns influenced by Chile's unique topography, integrating such data would not significantly enhance the predictive capabilities of our models. The topography remains relatively stable over short time periods, and its impact on air quality models would be limited. Instead, our focus has been on demonstrating the statistical relevance and accuracy of our models based on the available data.

**6. Potential Improvement: Hybrid Modeling Approaches
To address the limitations of the ARIMA model's reliance on autoregression and moving averages, the authors could explore hybrid modeling approaches that integrate multiple forecasting techniques. For example, ensemble methods combining ARIMA with machine learning algorithms such as ANN or gradient boosting machines (GBM) can leverage the strengths of each model while mitigating their weaknesses. By blending the predictive power of different methodologies, hybrid models may yield more robust and accurate air quality forecasts.**

The selection of ARIMA and ANN models was informed by their demonstrated effectiveness in time series forecasting, particularly in air quality prediction, aligning closely with the objectives of our study. Our rigorous validation process ensures that these models provide a robust fit to the data, yielding statistically significant parameter estimates and accurate predictions. While we acknowledge the existence of alternative modeling techniques such as ANFIS, Tree models, and optimization approaches, our decision to focus on ARIMA and ANN models was supported by their proven track record and compatibility with the available data. By integrating these two methodologies, we aimed to harness their complementary strengths in capturing both linear trends and nonlinear relationships inherent in air quality data. Our results underscore the competitiveness and suitability of ARIMA and ANN models for our research objectives while remaining open to exploring hybrid modeling approaches in future studies, should they offer demonstrable advantages in addressing specific research contexts and objectives.

**7. Comparison with the state-of the art literature is necessary, some of them are given as follows . https://ieeexplore.ieee.org/abstract/document/10363642 ; b. https://link.springer.com/article/10.1007/s11869-015-0369-9; c.https://www.mdpi.com/2071-1050/13/21/12217; d. https://sensors.myu-group.co.jp/sm_pdf/SM3362.pdf
e. https://ieeexplore.ieee.org/abstract/document/10288482 .
While the integrated ARIMA and ANN models represent a valuable contribution to air quality prediction, addressing the identified limitations and exploring potential enhancements can further advance the research in this field. By incorporating spatially explicit modeling techniques and exploring hybrid modeling approaches, researchers can strive to improve the accuracy and reliability of air quality forecasts, ultimately contributing to better environmental management and public health outcomes.**

In comparing our integrated ARIMA and ANN models with the state-of-the-art literature, it is essential to acknowledge the diverse approaches and methodologies employed in air quality prediction research. While the articles provided offer valuable insights into various aspects of air quality modeling and forecasting, it is noteworthy that only a subset directly relates to our specific study. Nevertheless, each of these references contributes to the broader discourse on air quality management and prediction. For instance, the study referenced in the first link (https://ieeexplore.ieee.org/abstract/document/10363642) highlights the use of low-cost sensors and incorporates altitude as an input parameter for air quality models. While our study leverages data from the extensive SINCA network in Chile, which comprises numerous monitoring stations across different regions, including Quintero and Coyhaique, we recognize the importance of considering alternative data sources and sensor technologies to enhance model accuracy and coverage. Similarly, the article cited in the third link (https://www.mdpi.com/2071-1050/13/21/12217) explores the application of machine learning algorithms for air quality forecasting, emphasizing the role of predictive modeling in supporting environmental policies. Our research aligns with this objective, as we aim to provide long-term air quality forecasts that inform public policy decisions and interventions aimed at improving environmental quality and public health outcomes. It is crucial to emphasize that while our study focuses on long-term air quality forecasting, which is pertinent for informing strategic policy decisions, the articles referenced by the reviewer offer valuable insights into alternative modeling approaches and considerations for future research directions. By incorporating insights from these studies into our discussion of results, we enrich the overall discourse on air quality modeling and forecasting, further advancing the field towards more robust and comprehensive predictive capabilities.
